# The Role of C-reactive Protein Estimation in Determining the Duration of Antibiotic Therapy in Neonatal Sepsis

**DOI:** 10.7759/cureus.30211

**Published:** 2022-10-12

**Authors:** Partha Kumar Chaudhuri, Ananya Ghosh, Vivek Sinha, Bhuwan Kumar Singh, Manisha Singh, Halyna Lugova, Rahnuma Ahmad, Susmita Sinha, Mainul Haque, Santosh Kumar

**Affiliations:** 1 Paediatrics, Rajendra Institute of Medical Sciences, Ranchi University, Ranchi, IND; 2 Humanitarian Assistance and Disaster Relief Research Centre, Universiti Pertahanan Nasional Malaysia (National Defence University of Malaysia), Kuala Lumpur, MYS; 3 Physiology, Medical College For Women and Hospital, Dhaka, BGD; 4 Physiology, Khulna City Medical College and Hospital, Khulna, BGD; 5 Pharmacology and Therapeutics, Universiti Pertahanan Nasional Malaysia (National Defence University of Malaysia), Kuala Lumpur, MYS; 6 Periodontology and Implantology, Karnavati University, Gandhinagar, IND

**Keywords:** serious infection, marker of inflammation, acute inflammation, newborn baby septicopyemia, newborn infant, septicemia, antimicrobial treatment, assessment, c-reactive protein, function

## Abstract

Introduction: Septicemia is globally considered the most important cause of neonatal morbidity and fatality. Serum C-Reactive Protein (CRP) is an acute phase reactant, which is brought out in response to the inflammatory reaction. It is prophesied to drop down speedily after the coherent weeding out of microbial incitation due to the short half-life of CRP. CRP levels reflect the individual's association between microbial infection and defensive mechanisms.

Methods: This hospital-based cross-sectional study included 150 admitted patients with suspected sepsis in the Department of Pediatrics, Rajendra Institute Medical Sciences (RIMS), Ranchi, India, over a study period of one year (2020 to 2021). CRP was estimated on the day of admission and repeated after 72 hours, on the fifth day, and on the seventh day for serial values of CRP, and the findings were compared by making three groups. Further, the research participants were designated to three different groups according to the CRP estimation levels.

Results: Out of the 150 assumed neonatal septicemia patients, antibiotics were paused in 42 neonates (28%) within 72 hours. In group 2, 8% of neonates’ antibiotics were stopped in five days, and a total of 102 neonates (68%) could be discharged on the seventh day of antibiotic therapy as their CRPs became negative on the third day and seventh day consecutively, along with negative blood culture reports. In group 3, antibiotics of 48 neonates (32%) were continued beyond seven days.

Conclusion: CRP has a skyscraping specificity and negative predictive values (NPV); thus, by estimating serial CRPs, the antibiotic therapy duration can be determined, which further helps determine the period of hospitalization.

## Introduction

Globally sepsis is one of the most typical causes of newborn mortality and fatality [1]. According to the National Neonatal Perinatal Database (NNPD), 2002-2003, episodes of neonatal sepsis (NS) in India is seen in 30 per 1000 live births [2]. Worldwide, approximately 3,000,000 infants per year suffer from NS (2202/100,000), and India has a very high level of occurrence of clinical sepsis (17,000/100,000 live births) [3]. NS is a clinical syndrome described as a microbial infection in the bloodstream of a neonate in the presence of a fever. It is a life-threatening condition in the initial 28 days of an infant [4,5]. It comprises varying clinical manifestations including septicemia, pneumonia, meningitis, osteomyelitis, and urinary tract infections [6].

NS is a most conventional problem associated with considerable mortality and morbidity. Most neonates present with atypical symptoms of NS, and others mimic the symptoms, making it difficult to differentiate between them. Hence, distinguishing the infants who have NS from those with suspected sepsis becomes challenging. The most common diagnostic method is the blood culture, also thought about as the touchstone, but the demerit is that it takes at least 48-72 hours for the results to be available [7]. Moreover, the positive findings of blood culture are low and are afflicted by the blood volume inoculated, level of bacteremia and laboratory capableness, and most importantly, prenatal antibiotic use [8]. In economically backward countries, culture-negative sepsis, and treatment delay are responsible for most episodes [7].

Blood cultures frequently identify pediatric pathogens within 48 hours. Parents are often unaware of the early signs of NS and only take their infant to the hospital when they are incredibly ill; thus, the doctors have already lost precious time. Moreover, doctors need to wait 72 hours for the final results of the blood culture for the confirmed diagnosis. In such situations, doctors frequently do not tend to wait due to the situation's urgency. Hence, despite not knowing the exact diagnosis, they prescribe antibiotics. According to several studies, 11-23% of infants were pharmacologically intervened imprudently for sepsis [9,10]. This results in antibiotic resistance and many other short-term complications.

Moreover, different range of microorganism causes a diverse continuum of infection. So, in lieu of adhering to the standard protocol for the antibiotic duration, it should be according to causative microbes. Thereafter, there is an urgent necessity to scan for quick biomarker evaluation for NS, instead of holding back for long-duration blood culture-sensitivity findings.

Multiple earlier studies revealed that CRP levels might help decide antibiotic treatment duration [11-13]. CRP, among others, is considered the most studied and primarily used laboratory test for determining NS [14]. Serum CRP, an acute phase reactant, is generated within the hepatic system in just a few hours in reaction to inflammation and may rise to thousand folds as a critical response [15]. CRP level tends to fall quickly immediately after the effectual eradication of microbial stimulus. This is due to its brief half-life of 19 hours [16]. This leads to CRP levels reflecting the distinctive balance between the microorganisms and the immunological status of the neonate for determining the efficacy of antibiotics and thus providing guidelines regarding the duration of antibiotic therapy [16-18].

Since its clinical manifestations vary in a wide range from subtle to specific, the diagnosis and management of NS are challenging. This further results in widespread, prolonged, and unnecessary use of antimicrobials leading to the problem of antimicrobial resistance. The perfect (best) quality test for recognizing NS is blood culture, which becomes reliable only when performed diligently [19]. A careful evaluation of duration and treatment indications is the need of the hour. This will reduce the cost and duration of hospital stay, diminishing the chances of new antibiotic-resistance bacteria.

Serial CRP estimations for establishing or excluding the diagnosis of septicemia in neonates have existing evidence. Serial measurements are always put forward rather than a single value [20-22]. Thus, the aim of the current study was to ascertain serial CRP levels to assess inflammatory status. Subsequently, we can terminate antimicrobials at the proper time, so that it could ensure safe and effective therapeutic intervention of NS.

## Materials and methods

Study population

This study was conducted among 150 out of 627 suspected NS cases for one year in the newborn intensive care unit (NICU), Department of Pediatrics and Neonatology, Rajendra Institute Medical Sciences, Ranchi, Jharkhand, India, from June 2021 to May 2022. Neonates with a birth weight > 1500 grams, with speculation NS and started on empirical antibiotics based on signs and symptoms, were included in the study. Neonates less than 28 days old diagnosed with meningitis and requiring a longer duration of antibiotics, those admitted with birth asphyxia and extreme prematurity, those who underwent surgical intervention due to microbially-contaminated wounds, and neonates who have taken antibiotics previously were excluded.

This study obtained ethical assent from the Institutional Review Board of Rajendra Institute Medical Sciences (RIMS/IRB/2021/0123; dated: January 27, 2021). The study was conducted in the spirit of the Declaration of Helsinki (2008). Additionally, the parents of the participants were given informed details about the study, later to be published for the improvident of science, and written informed consent was obtained from the parents of the enrolled infants.

Clinical and laboratory investigations

All the cases registered for the study were interrogated for detailed history. They were clinically examined thoroughly and investigated. Blood samples were collected from 627 medically speculated NS pediatric patients hospitalized at the Rajendra Institute of Medical Sciences. All necessary clinical details were documented in the pre-designed proforma.

NS was provisionally determined on the basis of “sepsis score” [23], which includes symptoms and signs such as lethargy, refusal to feed, vomiting, weak cry, convulsions, diarrhea, apnea, tachypnea, hypothermia, fever, decreased capillary refill time, and umbilical ooze.

In the absence of clinical symptoms, NS diagnosis was based on the "perinatal infection risk score" [24], which includes foul-smelling amniotic fluid, unhygienic vaginal examination by healthcare workers, duration of labor exceeding 24 hours, birth asphyxia (Apgar <6 at one minute), birth weight 2.5 kgs or less, gestation age below 37 weeks, the time span of rupture of amniotic membrane 24 hours before delivery, maternal fever increases the possibility of septic cases. A sepsis score of over 4 demands further detailed investigation.

Newborns with suggestive characteristics of NS had a septic score over 4 and were screened using CRP and various hematological parameters with a predetermined cut-off value. Simultaneously, a blood culture was sent for investigation. Other investigations were taken based on relevant clinical situations, such as cerebrospinal fluid (CSF), urine analysis, and swabs from the focus of infection.

All newborns underwent sepsis screening per the department's standard protocol, which includes (i) total leukocyte count (TLC): Leucopenia, i.e., abnormal TLC of <5000/cu.mm; (ii) absolute neutrophil count (ANC): low counts per Munroe's chart for term infants and Mouzinho’s chart for preterm infants [25]; (iii) immature/total neutrophil >0.2, i.e., band cells count more than 20%;.(iv) micro-erythrocyte sedimentation rate (ESR) 15 mm in first hour; and (v) CRP >1mg/dl.

The blood samples were collected from the peripheral vein maintaining asepsis before administration of any antibiotic therapy. All cases were managed with standard protocol for neonatal septicemia, and antibiotics were started after sending a blood culture. Blood samples were collected immediately after hospitalization and repeated every 72 hours: third day, fifth day, and seventh day for serial estimation of CRP. Their findings were compared accordingly by making three groups. CRP was determined using an auto-analyzer machine (Microsemi CRP LC-667G, Horiba, Kyoto, Japan).

Other investigations such as liver function tests, CSF examination, abdominal x-ray, assessment of kidney physiology, umbilical ooze specimen for culture and sensitivity (C&S), CT scan of the brain, a vaginal specimen of the mother for C&S, stool routine examination, serum electrolytes, complete blood count, urine microscopy, prothrombin time, and partial thromboplastin time was conducted based on the clinical need.

Study groups

Infection Unlikely: Group 1

This batch comprised 313 neonates with CRP levels below 1 mg/dl at 72 hours after initiating antibiotics. A total of 50 patients were randomly selected out of these 313 patients. Computer-generated codes did the randomization. Antibiotics were discontinued as they were clinically stable, and negative blood culture was reported. Those who were still clinically not stable or symptomatically did not improve were continued with a longer duration of antibiotics.

Infection Likely: Group 2

If CRP remained above 1 mg/dl even after 72 hours of antibiotic therapy, neonates were included under the "Infection Likely" group (Group 2). This batch comprised 182 neonates with CRP levels above 1 mg/dl at 72 hours and below 1 mg/dl on the fifth day. A total of 50 patients were randomly selected. Computer-generated codes did the randomization. CRP was estimated every alternate day in this group, and antibiotics were stopped whenever CRP was less than 1 mg/dl. Here antibiotics were stopped for those neonates whose CRP level came <1mg/dl on the fifth day. Among them, those who were still clinically not stable or symptomatically did not improve were continued with a longer duration of antibiotics.

Group 3

**Figure 1:**
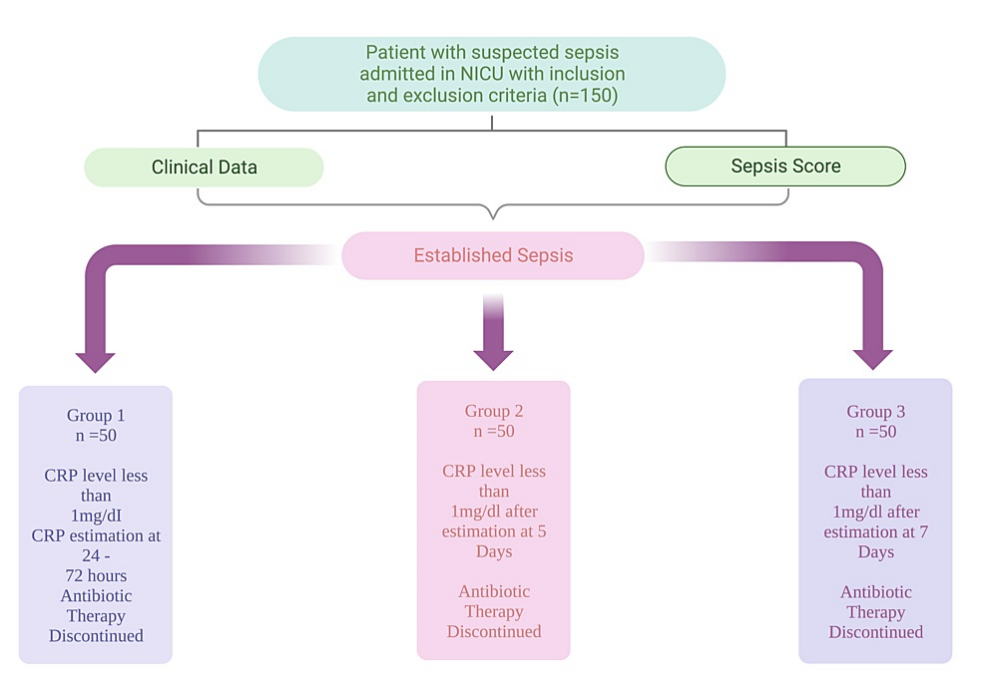
Flow chart denoting the research plan CRP: C-reactive protein; NICU: neonatal intensive care unit Image created with BioRender.com

This batch comprised 132 neonates with CRP levels above 1 mg/dl after the fifthday and less than 1 mg/dl on the seventh day. A total of 50 patients were randomly selected out of these 132 patients. Computer-generated codes did randomization. In this subgroup, antibiotics were continued from the first day till the seventh day. CRP was further measured on the 7th day. If CRP was below 1 mg/dl and the neonate had no symptoms, antibiotics were paused unless decided otherwise. If clinically, the neonate did not show resolution of symptoms even though CRP was negative, a decision to give antibiotics for a longer duration was taken. Neonates with blood culture positive reports were also given a more extended period of antibiotics on basis of a culture report (Figure 1).

Follow-up

Follow-ups of all the newborns were continued up to four weeks post-stoppage of antibiotics after observing the clinical signs of septicemia. The study groups were further rearranged into "relapse" and "no-relapse" groups.

No Relapse

Complete absence of any symptoms and signs of septicemia within four weeks of ooze.

Relapse

If the infant needs further antimicrobial therapy for assumed/proven septicemia within 28 days of oozing.

Sample for blood culture

One ml of blood was collected from the median cubital vein by maintaining all the asepsis protocols. It was then passed on to the container for blood culture media with 5 ml of brain heart infusion (BHI) broth and dispatched to the experiment room for additional procedure. Blood cultures were closely monitored for at least 72 hours before they were turned up as sterile.

CRP estimation

Blood sampling was done from the peripheral vein. Two ml of blood were collected under aseptic conditions from all admitted neonates with suspected sepsis and tested for various parameters. An auto-analyzer machine (Microsemi CRP LC-667G) was used for CRP determination. Test tubes of samples were loaded into racks, and the racks were inserted directly into the analyzer machine. It is a reliable, relatively more straightforward, and less time-consuming method for CRP determination.

Statistical methods

Standardized forms (case record forms) were used to record the relevant history, clinical examination findings, and laboratory data for admitted neonates with suspicion of sepsis before uploading them to a database maintained to track the clinicopathological progress of the neonates. Data was collected, recorded, and compiled on a Microsoft Excel data sheet (Microsoft Corporation, Redmond, Washington, United States). A conclusion was drawn by assessing the sensitivity, specificity, positive predictive value (PPV), and negative predictive value (NPV) of CRP as clinical parameters. Data were analyzed with appropriate statistical tests and methods to determine the study's significance and power. Descriptive statistics were expressed in a ratio, proportion, or percentage (for categorical data) and mean with standard deviation, median, or range (for numerical data). Chi squire test was used for analytical statistics, Fischer’s test for categorical data, student t-test paired and unpaired for parametric data, Wilcoxon’s test, and Mann Whitney’s test for nonparametric data, as applicable. Data were analyzed using IBM SPSS Statistics for Windows, Version 21.0 (Released 2012; IBM Corp., Armonk, New York, United States).

## Results

Patients demographics

This observational study was carried out to ascertain the duration of antibiotic therapy by sequential CRP findings conducted in the NICU, Department of Pediatrics. This study included 150 neonates with suspected septicemia and was studied for over one year. Of 150 patients, 93 were males, and the remaining 57 were females. The mean birth weight was 2.2 kg. Seventy-three percent of the subjects suffered from early-onset sepsis, whereas the remaining 27% suffered from late-onset sepsis. Maternal infections and positive blood cultures were present in 83% and 18% of cases, respectively. The mean duration of antibiotic therapy was 7.46 days (Table 1).

**Table 1:** Demographic characteristics of the patients

Total Number of patients	
Mean birth weight	2.20 ± 1.62 (KG)
Males	93 (62%)
Females	57 (38%)
Early onset sepsis	109 (73%)
Late-onset sepsis	41 (27%)
Positive blood culture	27 (18%)
Maternal infections	83%
Mean duration for antibiotic therapy	7.46 ±2.5 (days)

Seventy-three percent of cases were reported within 72 hrs of birth, whereas 27% of patients presented after 72 hours of delivery. Among the ones who presented after 72 hours, 22 cases presented between four to seven days of birth, 10 showed within 7-14 days, and nine presented within 15-28 days of birth. Of the cases, 62% were male and 38% were female, evenly disseminated among the three groups (Table 2).

**Table 2:** Duration of onset of sepsis in new-born

Within 72 hours of early-onset sepsis (EOS)	>72 hrs (late-onset sepsis (LOS))		
	4-7 days	7-14 days	15-28 days
109 (72.6%)	22 (14.6%)	10 (6.6%)	9 (%)

About 56% of the cases weighed below 2 kg, contributing to more than half of the patients. Nearly half of the cases, about 44%, constituted neonates weighing between 2-3.5 kg (Table 3). Most of the neonates (58%) were preterm, and the rest were term (38%) and post-term (4%). An equal number of patients had a history of the risk factor of maternal fever (41%) and premature rupture of membrane (PROM) (42%). A maximum number of subjects were presented with grievances of poor feeding (96%), lethargy (72%), tachypnea (52%), poor cry (48%), fever (28%), and excessive cry (18%).

**Table 3:** Distribution of neonatal sepsis according to weight (a) and maternal infection (b). Distribution of newborns based on symptoms (c). PROm: prolonged rupture of membranes

Group	Weight	Distribution	Percentage
a	1.5-2kgs	84	56%
	2-2.5 Kgs	18	12%
	2.5-3 Kgs	27	18%
	3-3.5 Kgs	21	14%
b	Symptoms	Distribution	Percentage
	Maternal fever	62	41%
	PROM	63	42%
c	Symptoms	Distribution	Percentage
	Poor feeding	144	96%
	Tachypnea	78	52%
	Poor cry	73	48%
	Fever	42	28%
	Lethargy	108	72%
	Excessive cry	26	18%

**Figure 2:**
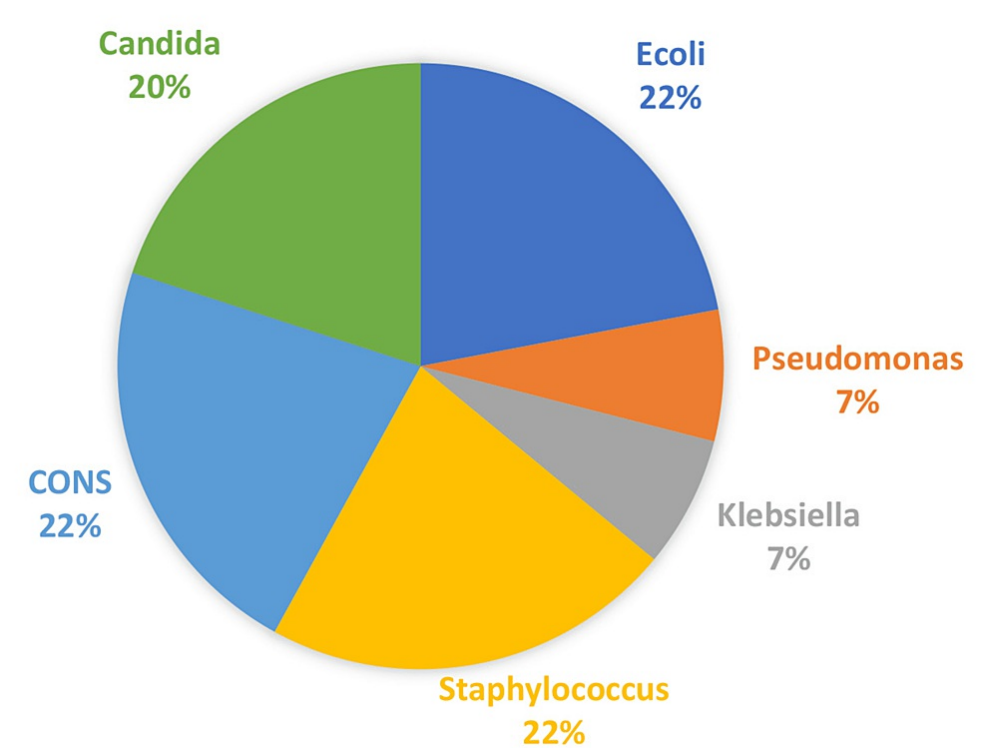
Distribution of neonatal sepsis based on culture reports CONS: coagulase-negative staphylococci; Ecoli: *Escherichia coli*

Culture analysis showed 18% of cases with positive blood cultures. Maximum blood culture reports were positive in Group 3, which is 16%, and none of the blood culture reports were positive in Group 1. Among 27 culture-positive cases, 17cases (62.9%) grew gram-positive organisms, and 10 (37%) cultures showed gram-negative microorganisms. Among gram-positive organisms, *Staphylococcus *(22%), coagulase-negative staphylococci (CoNS) (22%), and *Candida* (20%) were isolated in almost equivalent numbers, whereas among gram-negative organisms, it was *Escherichia coli* (22%) that was commonly isolated (Figure 2).

Group 1 consisted of 42 cases out of 150 patients with suspected sepsis. CRP levels decreased considerably after 72 hours, and antibiotics were paused. Those neonates who were still clinically not stable or symptomatically did not improve were continued with a longer antibiotic duration. But there were eight cases of relapse in the remaining four weeks (Table 4).

**Table 4:** CRP-guided distribution of cases CRP: C-reactive protein

CRP value	Groups	Duration of therapy	No. of cases	Blood culture positive	Relapse	Negative predictive value (NPV)
< 1mg/dl	Group 1	3 Days	42	Nil	8	80
		7 Days	8	Nil	Nil	100
>1 mg/dl	Group 2	5 Days	8	Nil	Nil	100
		7 Days	42	2	2	96
	Group 3	7 Days	2	NIL	NIL	100
		>7 Days	48	25	2	96

Group 2 consisted of 50 cases, out of which CRP normalized in eight cases on the fifth day, and antibiotics were stopped. Blood culture came out to be negative, and there was no relapse. In the rest of the 42 neonates, antibiotics were continued as CRP was elevated along with positive culture reports in two cases, and there were two relapses (Table 4).

Group 3 comprised 50 neonates, out of which CRP became negative on day seven of treatment in two cases, and antibiotics were stopped with no relapse. In maximum neonates in this group, antibiotics were continued for a duration longer than seven days as CRP remained positive or because of their blood culture reports, which turned out to be positive (Table 4).

**Figure 3:**
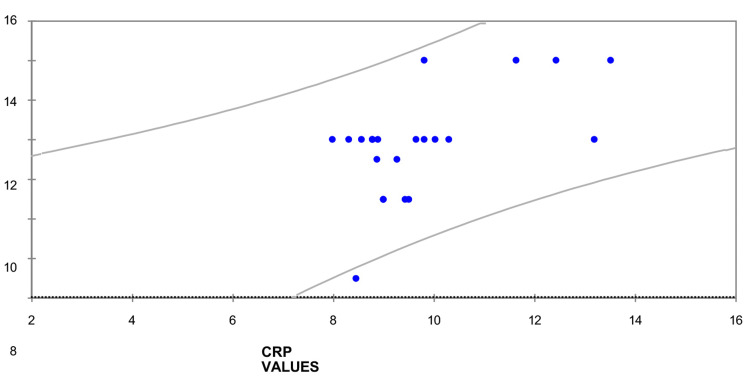
Linear regression curve of CRP and duration of antibiotic therapy. CRP: C-reactive protein

Out of the 150 suspected sepsis cases, antimicrobials could be halted within seven days in 102 cases (68%). In 42 patients (28%), antimicrobials could be ceased after full three days of initiation. Eight cases were given antibiotics for seven days. In group 2, antibiotics could be stopped in eight cases on the fifth day, and the remaining 42 cases received antibiotics for seven days. In Group 3, in only two patients, antibiotics could be stopped on the seventh day; in the remaining 48 cases, it was decided to continue antibiotics longer than seven days (Table 5). The linear regression curve (Figure 3) showed a positive correlation between the independent variable (CRP) and the dependent variable (length of antimicrobials therapies).

**Table 5:** CRP-guided overall duration of treatment CRP: C-reactive protein

Group	Duration of treatment				Total
	< 7 days			>7 days	
	3 days	5 days	7 days		
Group 1	42		8		50
Group 2	--	8	42		50
Group 3	--	--	2	48	50
Total	42	8	52	48	150

## Discussion

**Figure 4:**
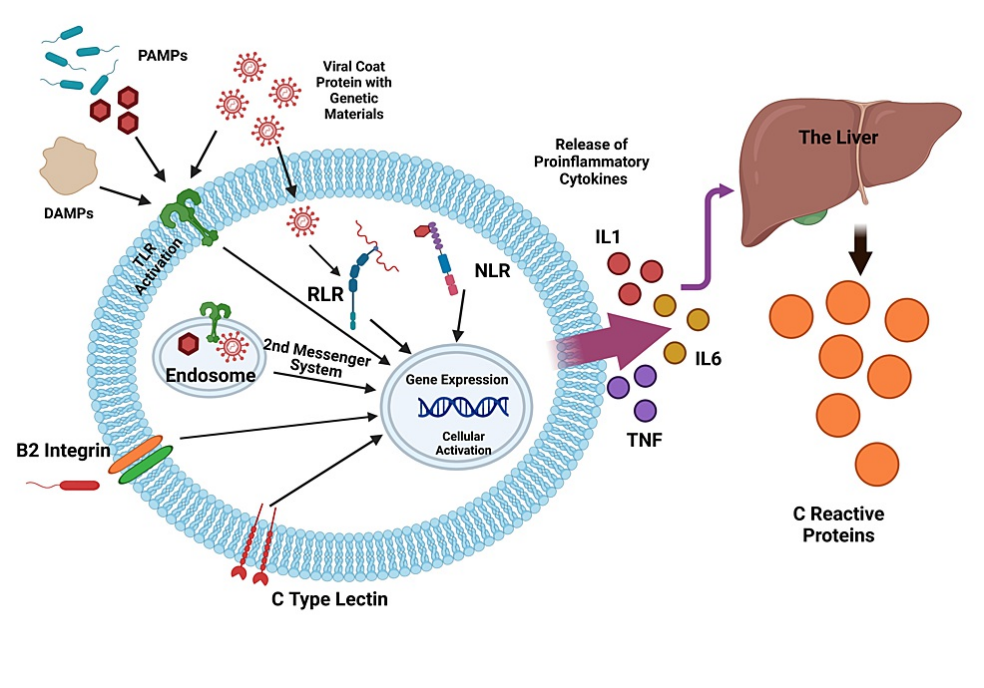
Activation of immune cells and release of C-reactive protein in neonatal sepsis PAMPs: pathogen-associated molecular patterns; DAMPs: damage-associated molecular patterns; TLR: toll-like receptor; RLR: retinoic acid-inducible gene I (RIG-I)-like receptors; NLR: nod-like receptor; TNF: tumor necrosis factor; IL: interleukin Image Credit: Susmita Sinha

The liver generates serum CRP in just a few hours of inflammatory reaction and often raises it to a thousand times in an acute case (Figure 4) [15]. NS is one of the significant reasons for neonatal mortality in developing countries leading to a major health problem [26]. Diagnosing infection during the neonatal period is crucial to managing this problem [27]. Globally, neonatal units are facing multiple obstacles regarding the alternating pattern of causative organisms; more importantly, there is widespread resistance to common antimicrobials by these organisms because of the over-jealous use of antibiotics and ineffective infection prevention and control [28]. So, measures are to be taken at every step to reduce the overuse of antimicrobials and limit the emerging resistance. In the current study, an effort has been made to know the various etiological agents responsible for NS and correlate the efficacy of the sepsis score and the sepsis screening parameters like CRP in estimating and reducing antibiotic therapy duration.

In the present study, NS was higher among males (62%) than females (38%), with a ratio of 1.6:1. These results are comparable to similar earlier studies [18,29-31]. The incidence of early-onset sepsis (EOS) was 73%, and late-onset sepsis (LOS) was 27% in the present study. This study concurred with the NNPD Report (2002), which found 67% and 33% of EOS and LOS, respectively [2]. However, the study by Varsha et al. showed a different aspect where LOS was seen in 52% and EOS was seen in 48% [32].

In the present study, most cases had preterm gestation (58%) compared to term gestation (38%). This was inconsistent with other studies, as given by Khatua et al., in which 63% of the total cases were preterm [33]. In their study, Joshi et al. also had similar results, where 52% were preterm and 48% were term [29]. In this study, lethargy, poor feeding, and tachypnea were the most typical signs and symptoms, along with fever and excessive crying. Khinch et al. observed similar results where poor feeding, tachypnea, and high body temperature were the most typical symptom, followed by weak cry, pyrexia, and yellowish or greenish pigmentation of the skin and sclera [34].

**Figure 5:**
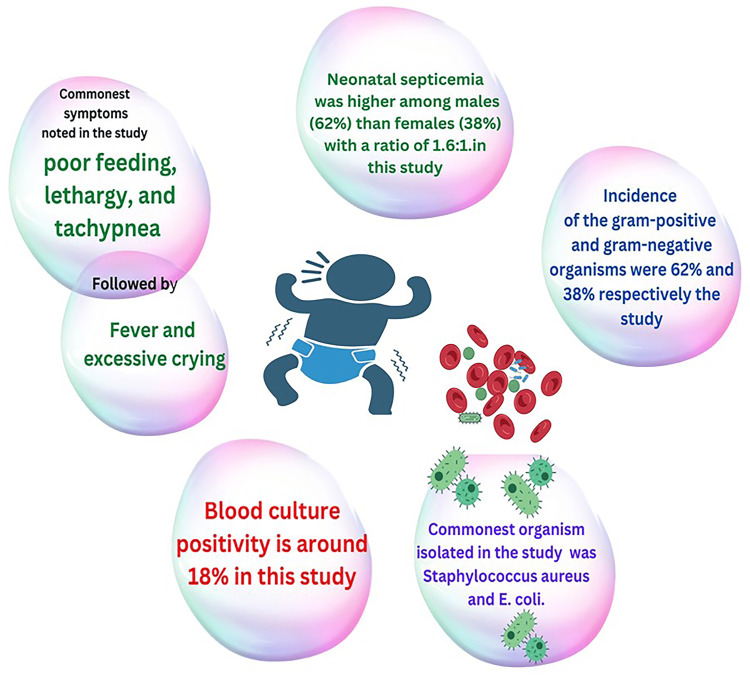
Summary of the different findings of the study regarding gender difference in incidence for neonatal septicemia, symptoms, most common bacteria, and blood culture findings Image Credit: Rahnuma Ahmad

Positive blood culture in this study is reported to be 18%, which concurs with the research carried out by Joshi et al. [29] and Shrestha et al. [35], in which incidence was 25% and 34%, respectively. A different study by Tallur et al.showed a considerably greater incidence (65%) of positive blood culture [36]. The incidence of the gram-positive and gram-negative organisms were 62% and 38%, respectively. The most typical organism isolated among them was *Staphylococcus aureus* and *E. coli*. Shaw et al. revealed that isolated* Staphylococcus* and *Klebsiella* were the most frequently involved pathogens [37]. There are other studies with similar positive blood culture reports [38-40]. Some studies mentioned above, however, showed a higher incidence of the gram-negative organism [38,40-42]. Figure 5 summarizes the findings of gender differences in NS incidence, symptoms, blood culture, and bacteria in this study.

Comparison of CRP-guided therapy groups

The current study showed that antimicrobials were paused in Group 1 in 42 (28%) neonates after 72 hours of pharmacological intervention, and there were eight relapse patients found in the next four weeks. In one study, antimicrobials were discontinued in 48% of patients [43], and in another study, antimicrobial therapy was terminated in 44% of cases within 72 hours of starting the treatment [30]. In a similar study, antibiotics were halted in 32 (82.5%) neonates within 72 hours [44]. As mentioned above, NPV in the studies was 99% [45], 100% [43], and 86% [7], which is greater than the recorded data of 80% in the present study. A separate study revealed that antibiotics could be stopped in 38% of cases in less than two days, depending on CRP estimation [30]. No relapses were found after that, with an NPV of 100%, which is even higher than the current study.

In Group 2, in eight out of 50 patients, antimicrobials could be paused on the fifth day, and in the remaining 42 cases, CRP levels remained raised. Thus, antibiotics were continued for up to seven days in those cases. There were two relapses in this group, giving 96% as an NPV compared to the earlier study [43], which showed that 38 out of 39 cases (97.8%) were guided by CRP within six days with an NPV of 99%.

In Group 3, only two out of 50 cases of antibiotics therapy could be terminated, thus assigning an NPV of 100% to this group. In 48 patients, treatment was carried on for a further seven days as CRP levels were pushed up. There were two relapses in this group, and NPV was 96%, similar to the earlier study, which showed serum CRP remained higher even after the fifth day of medication [43]. Two out of 48 cases had relapsed and required to repeat dosing of antimicrobials within four weeks, giving an NPV of 96%. Multiple studies revealed 100% in both studies [46,47]. Antimicrobials were discontinued in two out of 50 patients in less than seven days in our study.

The present study has limitations as the overall results of our study are valid for the specified group of neonates with gestational age (GA) >32 weeks and birth weight ≥1.5 kg without central catheters. Thus, the extent of applauding blood cultures in the present exploration was comparatively minimum. Moreover, the findings are constructed on a specific combination of antimicrobials used in the study and a particular pathogen common in this hospital. Thus, the interpolation of the findings of the current study for other hospitals should be based on careful clinical and medical reports of that healthcare premises. The recurrence percentage in CRP-guided therapy (Group 2) and one-week treatment (Group 3) can be explained because of the relatively small sample size of this investigation. The incidence of positive blood culture is relatively low as we have excluded very low birth weights, and neonates using incursive substances were excluded.

## Conclusions

In the quantitative method carried out for CRP estimation, a rapid, simple, and inexpensive test was found to have high sensitivity, moderate specificity, and high NPV. The NPV of serial serum CRP accounts for 98-100% in deciding the period of antimicrobial intervention in NS for up to a full week. Thus, the duration of antimicrobial medication could be reduced to below one week and three days among 68% and 28% of patients, respectively, as the current study has a negative CRP result. Newborns with suspected sepsis with positive blood culture and raised CRP needed a longer duration of antibiotic therapy. CRP may, thus, help in the earlier recognition or diagnosing of NS whilst waiting for a blood culture report and lead to a subtle time reduction of the treatment, which in turn leads to judicious use of antibiotics undergoing CRP-guided therapy, making it one of the feasible parameters. Additionally, CRP recording at serial intervals could help modify antibiotic regimens accordingly and help manage the cases, thus shortening the hospital stay of neonates and preventing the emergence of resistance, decreasing treatment costs, reducing adverse effects, and less interference with the microbiome.
